# Read-Split-Run: an improved bioinformatics pipeline for identification of genome-wide non-canonical spliced regions using RNA-Seq data

**DOI:** 10.1186/s12864-016-2896-7

**Published:** 2016-08-22

**Authors:** Yongsheng Bai, Jeff Kinne, Brandon Donham, Feng Jiang, Lizhong Ding, Justin R. Hassler, Randal J. Kaufman

**Affiliations:** 1 Department of Biology, Terre Haute, USA; 2 The Center for Genomic Advocacy, Indiana State University, 600 Chestnut Street, Terre Haute, IN 47809 USA; 3 Department of Mathematics and Computer Science, Indiana State University, 200 North Seventh Street, Terre Haute, IN 47809 USA; 4 Sanford-Burnham-Prebys Medical Discovery Institute, La Jolla, California 92037 USA

**Keywords:** Alternative splicing, Non-canonical, RNA-Seq, *Xbp1*, ENCODE

## Abstract

**Background:**

Most existing tools for detecting next-generation sequencing-based splicing events focus on generic splicing events. Consequently, special types of non-canonical splicing events of short mRNA regions (IRE1α targeted) have not yet been thoroughly addressed at a genome-wide level using bioinformatics approaches in conjunction with next-generation technologies. During endoplasmic reticulum (ER) stress, the gene encoding the RNase *Ire1*α is known to splice out a short 26 nt region from the mRNA of the transcription factor *Xbp1* non-canonically within the cytosol. This causes an open reading frame-shift that induces expression of many downstream genes in reaction to ER stress as part of the unfolded protein response (UPR). We previously published an algorithm termed “Read-Split-Walk” (RSW) to identify non-canonical splicing regions using RNA-Seq data and applied it to ER stress-induced *Ire1*α heterozygote and knockout mouse embryonic fibroblast cell lines. In this study, we have developed an improved algorithm “Read-Split-Run” (RSR) for detecting genome-wide *Ire1α*-targeted genes with non-canonical spliced regions at a faster speed. We applied the RSR algorithm using different combinations of several parameters to the previously RSW tested mouse embryonic fibroblast cells (MEF) and the human Encyclopedia of DNA Elements (ENCODE) RNA-Seq data. We also compared the performance of RSR with two other alternative splicing events identification tools (TopHat (Trapnell et al., Bioinformatics 25:1105–1111, 2009) and Alt Event Finder (Zhou et al., BMC Genomics 13:S10, 2012)) utilizing the context of the spliced *Xbp1* mRNA as a positive control in the data sets we identified it to be the top cleavage target present in *Ire1α*^*+/−*^ but absent in *Ire1α*^*−/−*^ MEF samples and this comparison was also extended to human ENCODE RNA-Seq data.

**Results:**

Proof of principle came in our results by the fact that the 26 nt non-conventional splice site in *Xbp1* was detected as the top hit by our new RSR algorithm in heterozygote (Het) samples from both Thapsigargin (Tg) and Dithiothreitol (Dtt) treated experiments but absent in the negative control *Ire1*α knock-out (KO) samples. Applying different combinations of parameters to the mouse MEF RNA-Seq data, we suggest a General Linear Model (GLM) for both Tg and Dtt treated experiments. We also ran RSR for a human ENCODE RNA-Seq dataset and identified 32,597 spliced regions for regular chromosomes. TopHat (Trapnell et al., Bioinformatics 25:1105–1111, 2009) and Alt Event Finder (Zhou et al., BMC Genomics 13:S10, 2012) identified 237,155 spliced junctions and 9,129 exon skipping events (excluding chr14), respectively. Our Read-Split-Run algorithm also outperformed others in the context of ranking *Xbp1* gene as the top cleavage target present in *Ire1α*^*+/−*^ but absent in *Ire1α*^*−/−*^ MEF samples. The RSR package including source codes is available at http://bioinf1.indstate.edu/RSR and its pipeline source codes are also freely available at https://github.com/xuric/read-split-run for academic use.

**Conclusions:**

Our new RSR algorithm has the capability of processing massive amounts of human ENCODE RNA-Seq data for identifying novel splice junction sites at a genome-wide level in a much more efficient manner when compared to the previous RSW algorithm. Our proposed model can also predict the number of spliced regions under any combinations of parameters. Our pipeline can detect novel spliced sites for other species using RNA-Seq data generated under similar conditions.

**Electronic supplementary material:**

The online version of this article (doi:10.1186/s12864-016-2896-7) contains supplementary material, which is available to authorized users.

## Background

In metazoans, during endoplasmic reticulum (ER) stress, the endoribonuclease (RNase) Inositol Requiring Enzyme 1a (*Ire1α*) initiates removal of a 26 nt region from the mRNA encoding the transcription factor *Xbp1* via an non-canonical mechanism (atypically within the cytosol). This causes a transitional open reading frame-shift to produce a potent transcription factor, *Xbp1s*, that induces expression of numerous downstream genes in response to ER stress as part of the unfolded protein response (UPR) [1, 2]. In addition, spliceosome-independent cytoplasmic splicing, as a part of the unfolded protein response pathway, has been described in yeast [3] where *HAC1p* was found to be the sole splicing substrate of *Ire1*. The mechanism of *Ire1α*-mediated RNA-splicing is likely conserved in all eukaryotes as well [4].

In recent years, many popular methods have been developed to identify novel splice sites in RNA-Seq data, including TopHat [5] and Alt Event Finder [6]. A detailed review on the limitations of several other tools for identification of alternative splicing events (TrueSight [7], Splicing-Compass [8], and PASTA [9]) was described previously [10]. In short, indeed none of these existing tools were suitably designed for detecting the type of non-canonical sometimes called non-canonical splice sites generated by *Ire1α*-targeted *Xbp1* mRNA splicing. Given that non-canonical splicing events of short mRNA regions occurring within the cytosol have not yet been investigated using next-generation technologies at a genome-wide level, cutting-edge bioinformatics methods of detecting such targets are needed to quickly discover such splicing events in a patient-specific manner in order to derive future therapeutic value.

In order to supply the medical and scientific fields with such a tool we previously developed a novel bioinformatics pipeline method, named Read-Split-Walk [10] for detecting non-canonical, short, splicing regions using RNA-Seq data. We applied the method to ER stress-induced *Ire1α* heterozygous and knockout mouse embryonic fibroblast (MEF) cell lines to identify *Ire1α* targets of which the 26 nt non-canonical splice site in *Xbp1* was detected as the most prominent splice target by our initial RSW pipeline in heterozygous (Het) samples, not mapped in the negative control *Ire1α* knockout (KO) samples for both Thapsigargin (Tg) and Dithiothreitol (Dtt) treated experiments. In our previous study, we also compared the *Xbp1* results from our approach with results using the alignment program BWA [11], Bowtie2 [12], STAR [13], Exonerate [14] and the Unix “grep” command. Although our previous RSW method gave better results overall than the above-mentioned approaches, we realized that RSW’s running speed needed to be further improved in order to handle the massive amount of data in other experiments (human ENCODE project: https://www.encodeproject.org). In addition, we wanted to test, under different combinations of parameters, how and where reported spliced regions would differ. Therefore, we have designed a newer algorithm which we call “Read-Split-Run” (RSR) that can process RNA-Seq data in a more efficient manner with flexible parameters. We also proposed a linear regression equation under the assumption of the Generalized Linear Model for RSR parameters that can automatically predict the number of spliced regions given any parameter settings for a particular experiment.

We compared our RSR algorithm with the above-mentioned alternative splicing events detection tools using metrics of how each tool ranks *Xbp1* as the top cleavage target and its presence and absence in *Ire1α*^*+/−*^ and *Ire1α*^*−/−*^ MEF samples. We have also compared our RSR pipeline and other tools to process a human ENCODE dataset and reported their statistics of running performance and sensitivity (the number of spliced junctions identified).

## Results

### The web interface features of RSR

**Fig. 1:**
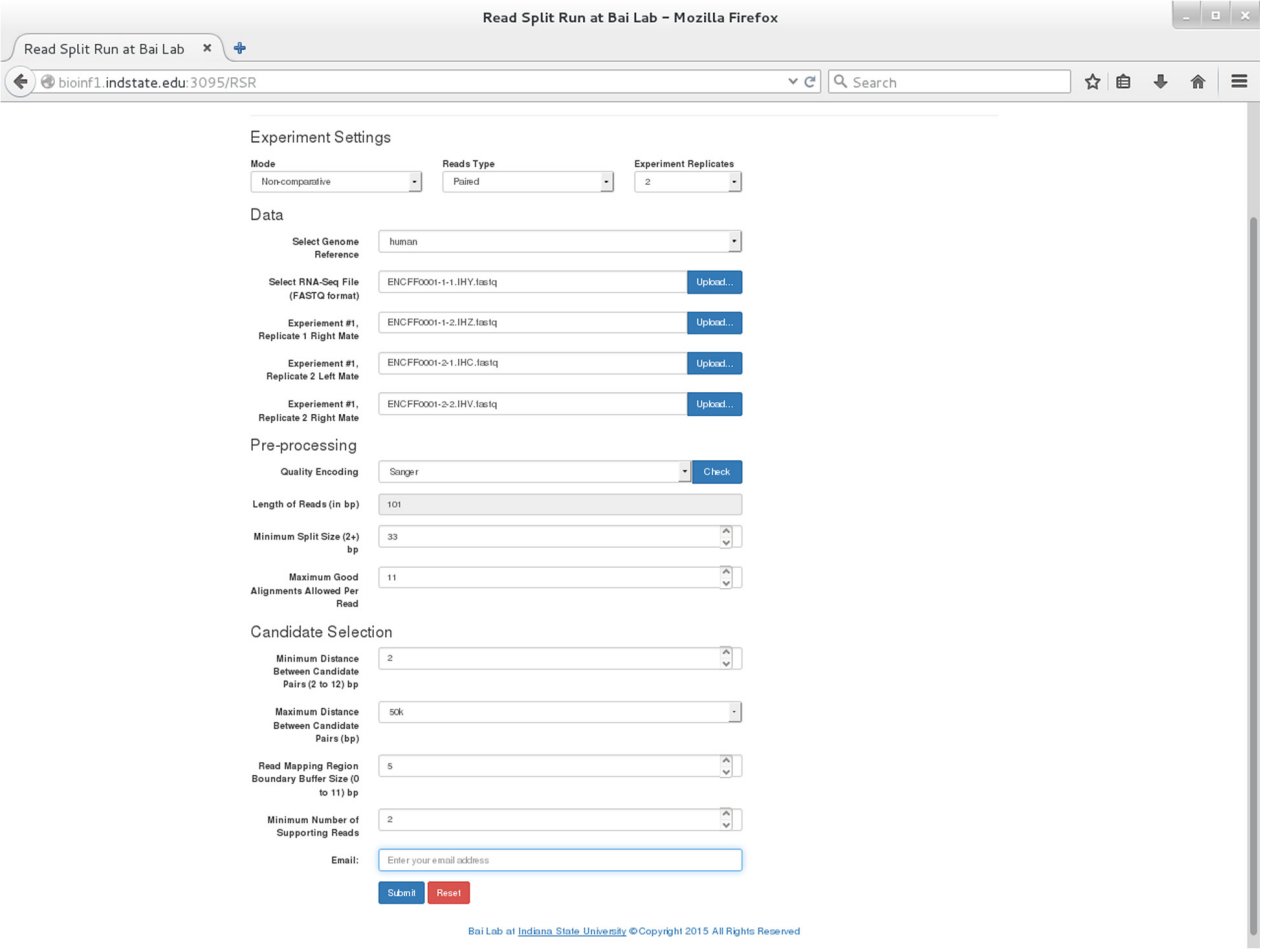
A screenshot for the Read-Split-Run web interface

In addition to providing the source code for download, the current web site of RSR (http://bioinf1.indstate.edu/RSR) allows users to use RSR by using a web form to upload data to the RSR server. After a job is submitted the server runs the pipeline and sends an email with a download link when the results are ready. The web form allows selection of a flexible combination of parameters. For example, users can select “Mode (Comparative or Non-comparative)”, “Reads Type (Single or Paired-end reads)”, “Experimental Replicates (1, 2, 3, …)”. The input files must be in FASTQ format. Based on the user’s initial selection, the pre-processing step will automatically reflect the number of input files needed. Users also have the options of checking the quality encoding and read length for short read input sequence files. The pipeline moves to the next step only if the read lengths for all input files are confirmed to be equal. A screenshot for the RSR web interface is shown in Fig. 1.

### The spliced regions detected by the RSR pipeline for mouse MEF RNA-Seq data

The identified spliced regions by the RSR pipeline for five cases with different combinations of parameters when processing *Ire1*α(+/−) and *Ire1*α(−/−) samples in both experiments (Tg and Dtt treated) are shown in Table 1. The detailed information of spliced regions identified by RSR for Tg and Dtt treated samples under different parameter settings are reported in Additional files 1 and 2. The attached Additional files 3 and 4 give statistics from running the new pipeline on mouse Tg and Dtt data with various parameter settings for MS, MD, and BB on a system with two Intel Xeon E5-2650 2.6GHz processors (a total of 16 cores and 32 hyperthreads).

**Table 1:** Comparison of total number of junctions identified by RSR for five cases from Tg and Dtt treated samples

	Parameter	Case 1	Case 2	Case 3	Case 4	Case 5
Variable Name	Minimum split size	**8**	**11**	**11**	**15**	**18**
	Maximum candidate distance	**40,000**	**40,000**	**50,000**	**50,000**	**50,000**
	Read mapping region boundary buffer size	**5**	**5**	**5**	**8**	**8**
	Minimum candidate distance	2	2	2	2	2
	Minimum number of supporting reads	2	2	2	2	2
	Maximum good alignment allowed per read	8	11	11	15	18
Tg Het	Total number of junctions identified	122	140	143	135	141
Tg KO	Total number of junctions identified	153	177	177	170	177
Dtt Het	Total number of junctions identified	6496	6614	6661	6673	5683
Dtt KO	Total number of junctions identified (Novel/Known)	6135	6247	6285	6308	5687

The bolded numbers show parameters with different test settings

### The spliced regions detected by the RSR pipeline for human ENCODE data

**Fig. 2:**
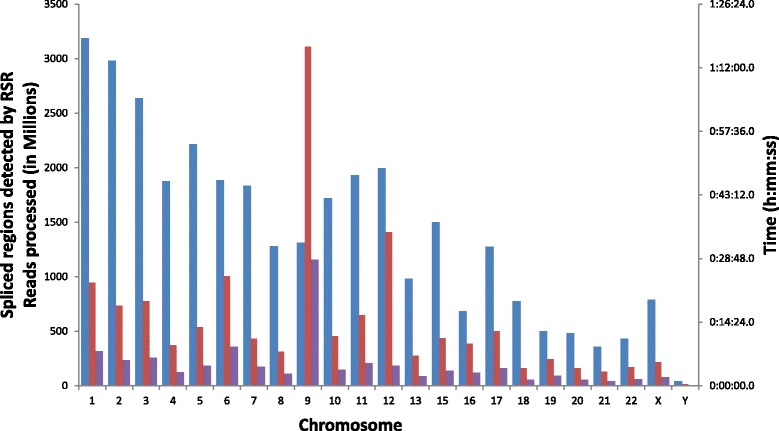
Number of junctions and clock time reported by RSR for the human ENCODE RNA-Seq sample (Separated by chromosomes). Blue bar: junctions; red bar: clock time; purple bar: reads processed (million)

The total number of spliced regions and running time of RSR on each chromosome of human ENCODE RNA-Seq data are shown in Fig. 2. The analysis for Chr14 was not performed due to memory constraints on the machine running the RSR pipeline. Additional file 5 shows the number of spliced regions identified by our RSR algorithm for each chromosome of the human ENCODE RNA-Seq dataset.

### Comparison of detected spliced regions between RSR and other tools

We also compared our RSR algorithm with other NGS based alternative splicing events detection tools (TopHat and Alt Event Finder). Our Read-Split-Run algorithm outperformed the other two software in the context of ranking *Xbp1* gene as the top cleavage target present in *Ire1α*^*+/−*^ but absent in *Ire1α*^*−/−*^ MEF samples. In particular, we have ran TopHat and Alt Event Finder on both Tg and Dtt samples of MEF cell line RNA-Seq data. TopHat identified 23 reads supporting *Xbp1* in Tg Het and 86 reads for Tg KO samples. For Dtt samples, TopHat reported a total of 59 (Het) and 289 (KO). Although the number of reads supporting *Xbp1* reported by TopHat in Tg Het sample is slightly higher than our RSR method (23 vs 21), our RSR accomplished a better turnaround (173) when compared to TopHat (59) in the Dtt dataset. Surprisingly, TopHat also reported reads supporting *Xbp1* in KO samples (86 in Tg and 289 in Dtt), which are false positive reads. Alt Event Finder did not identify any reads in supporting 26 nt *Xbp1* spliced regions. The comparison results between RSR and other tools in running RNA-Seq data from MEF cell line is listed in Table 2.

**Table 2:** Number of reads for supporting *Xbp1* 26 nt spliced regions reported by RSR and other tools

	500 nM Thapsigargin (Tg)		1 mM Dithiothreitol (Dtt)	
Software	Het (*Ire1α* ^+/−^)	KO (*Ire1α* ^−/−^)	Het (*Ire1α* ^+/−^)	KO (*Ire1α* ^−/−^)
Read-Split-Run (RSR)	21	0	173	0
TopHat	23	86	59	289
BWA	0	0	67	0
Bowtie2	0	0	171	0
STAR	0	0	0	0
Alt Event Finder	0	0	0	0

**Fig. 3:**
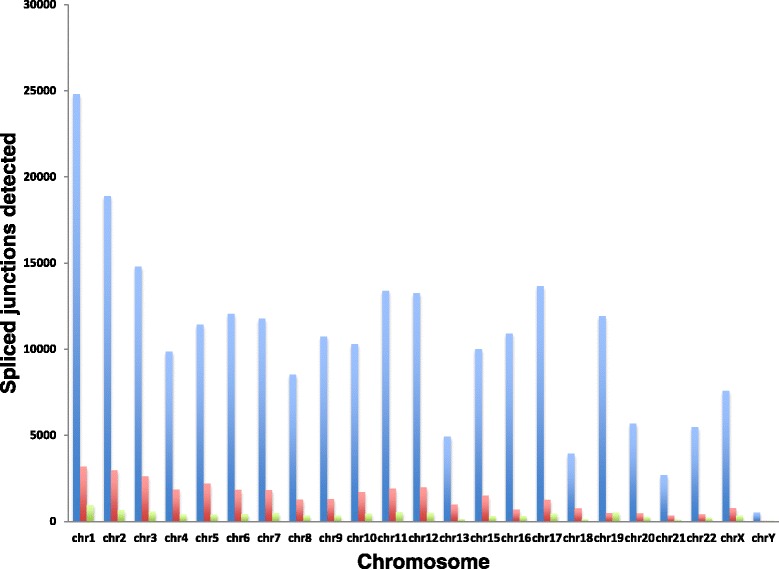
Comparison results in identifying number of spliced regions between RSR, Alt Event Finder, TopHat tools. Blue bar: TopHat; red bar: RSR; green bar: AltEventFinder

When applying these tools on human ENCODE RNA-Seq data, we found that the running speed and splicing regions detected by these tools are different. Due to the memory constraint of our server, we ran RSR by splitting the genome alignment files into individual chromosomes. It took RSR less than half an hour to run most chromosomes except for chromosome 9 and 12. In contrast, it took TopHat roughly 20 h to run the ENCODE RNA-Seq dataset. Alt Event Finder only required 3 h time to process the dataset, but the result was not informative for identifying short non-canonical spliced regions. Indeed, we have designed the web-based interface to and results reporting of our RSR pipeline to be as user-friendly as possible. Figure 3 shows comparison results between RSR and other tools in identifying number of spliced regions for the human ENCODE RNA-Seq dataset that we have tested.

### The general linear model for RSR algorithm

**Fig. 4:**
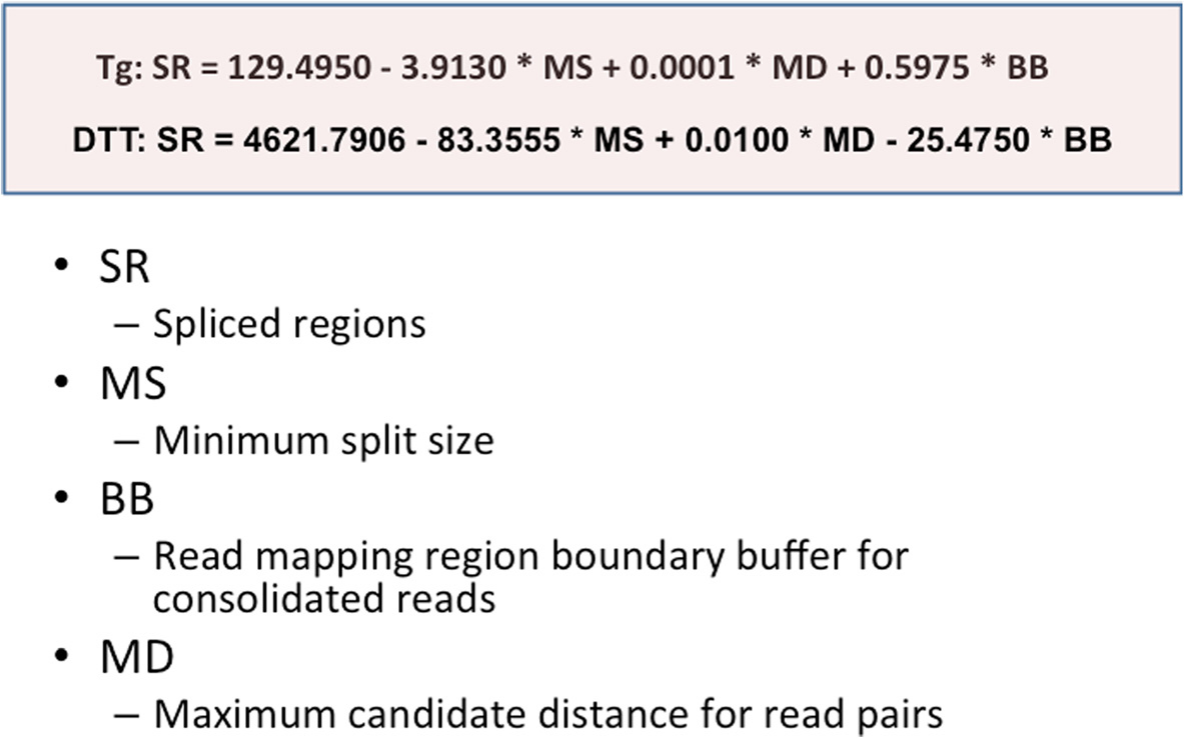
Linear regression equations for mouse MEF Tg and Dtt experiments

We generated a General Linear Model for both Tg and Dtt samples in the context of the total number of unique spliced regions (i.e. present in Het and not in KO samples) identified by the RSR algorithm (Fig. 4). The model is derived from the results based on many pairs of Het and KO test cases (100 for Tg and 64 for Dtt samples) with different combinations of three RSR algorithm parameters (minimum split size (MS), maximum candidate distance (MD), and read mapping region boundary buffer size (BB)). We therefore obtained two linear regression equations (one for Tg and the other for Dtt) as shown in Fig. 4. It is clear that the numbers of spliced regions identified by RSR decreases as MS values increase. This is true because the numbers of split pairs fed into the second step of bowtie decrease when MS values increase. The parameter of MD plays less critical roles as we expected. The BB parameter seems to follow the correlation (the numbers of spliced regions increase as BB values increase) for the Tg dataset, but not for the Dtt dataset. We would like to increase test cases for the Dtt sample to see whether the trend will change.

## Discussion

### Parameters consideration in RSR pipeline

Our proposed model of taking different parameter combinations to run the bowtie aligner and RSR algorithm could be applied to other species. However, different parameter combinations would predict different outcomes (i.e. number of spliced regions) for different species, even for different experiments. In our pipeline, we chose three parameters (MS, MD, and BB) to test how different combinations affect the prediction outcome. Specifically, we have run a number of test cases (100 for Tg and 64 for Dtt) with different combinations and used the results to generate linear regression equations. To increase the robustness of our RSR algorithm, it is ideal to perform a large-scale simulation study in order to look for the optimal combination. Typical questions remain to be answered: What would be the trade-off between lower/upper bound split size *vs* alignment sensitivity? What is the optimal consolidation slip/buffer size? More or fewer supporting reads will be reported if different cut-off criteria are applied, and these can be adjusted to achieve the desired balance between sensitivity and specificity in specific applications.

We chose three parameters (minimum split size (MS), maximum candidate distance (MD), and read mapping region boundary buffer size (BB)) for the RSR algorithm because the number of reads supporting spliced regions could be different given different combination values of these parameters. Our GLM was generated by running a number of test cases. A smaller MS and bigger MD and BB could increase the number of junctions reported. Empirically MS could be set to approximately 1/3 of the read length, and should also be larger than 8 bp to ensure the split half is not too short to be mapped accurately. An estimated MD value could be determined according to the average gene length of species tested. Therefore, users could choose their customized MD value according to the species on which their experiments were performed. This criterion is determined according to the assumption that the split pairs supporting the junction are often mapped onto the same gene. Finally, BB could be set to a number that should not reflect a large boundary variation (5 or less would be reasonable).

### The file deletion of our RSR algorithm

We noticed, by splitting reads into multiple read half pairs, the size of the result files substantially increased when the human ENCODE dataset was processed. To reduce the hard drive storage of large data files, we automatically delete files throughout the pipeline as they are no longer needed. For example, if the split step only needs to utilize the unmapped read datasets, the alignment files generated from the first step of bowtie are deleted. After the second step of bowtie is finished, all unmapped files will be deleted and only the alignment file will be kept for the next RSR step. The RSR pipeline can also compare spliced regions between samples and output reported regions side-by-side.

### RSR running speed, sensitivity, and specificity

In this study, we have developed a newer pipeline (RSR) of RSW with an improved running speed and proposed a General Linear Model for the algorithm. We used RSR to process different combinations of running parameters for the MEF and human ENCODE RNA-Seq data. We have compared our RSR algorithm with two popular NGS based alternative splicing events detection tools (TopHat and Alt Event Finder) and reported the spliced regions detection results. Neither of these two tools achieved better sensitivity (Number of junctions identified) than our RSR algorithm in identifying reads supporting the *Xbp1* 26 bp spliced region. This can be explained in part due to the fact that Alt Event Finder processes the mapped reads to report splice regions yet does not consider unmapped reads in the analysis input. Moreover, the current version of Alt Event Finder focuses on identifying exon skipping events only. TopHat reported canonical exon-exon splice sites as well. The spliced junctions identified by TopHat and our RSR are reported in Table 3. TopHat reported more junctions than our RSR. But many of them were known junctions or false positive ones. It is clear that the common junctions detected by both tools or overlapping rate is low. Indeed, the overlapping rate is even smaller for results from the human ENCODE dataset.

**Table 3:** The overlapping spliced junctions identified by TopHat and our RSR

	mouse-Het	mouse-KO	mouse-Het	mouse-KO	human
Software	500 nM Thapsigargin (Tg)		1 mM Dithiothreitol (Dtt)		ENCODE
TopHat	956	923	8897	7847	237,155
RSR	144	183	6727	6343	32,597
Common	38	41	2398	2128	314
Common/TopHat	3.97 %	4.44 %	26.95 %	27.12 %	0.13 %
Common/RSR	26.39 %	22.40 %	35.65 %	33.55 %	0.96 %

In our original RSW paper, we also compared the *Xbp1* results from our approach with results using the alignment program BWA [11], Bowtie2 [12], STAR [13], Exonerate [14] and the Unix “grep” command. Although our RSW method gave better results overall than the above-mentioned approaches, comparison results also suggested that reads supporting removal of the 26 nt intron from *Xbp1* mRNA were not fully acknowledged. A study using in vitro cleavage assay combined with microarray analysis reported 13 additional mRNAs as *Ire1α* cleavage targets [15]. The discovery shed light on the existence of other possible targets. A future version of the algorithm will focus on rescuing these false negative reads in order to achieve a better sensitivity.

### Applying RSR on human ENCODE RNA-Seq data

The discovery of a new set of non-canonical splicing events in humans is important not only because of the obvious potential for novel alteration of targeted transcript function, but also the potential for the resulting excised sequences to function as silencing RNAs associated with particular disease states. In addition, the frequency of these novel splicing events could be subject to altered regulation in some individuals, resulting in identifiable splicing profiles associated with the risk of certain diseases. We used ENCODE RNA-Seq datasets to train our RSR algorithm and hope to identify additional targets and elucidate their splicing patterns. Results should eventually provide unique insight of elucidating how short non-canonical spliced sequences act their biological functions in the context of relevant biological processes and diseases.

## Conclusions

The positive control for our application, the *Xbp1* 26 nt non-canonical splice site, was clearly detected in Het samples but not in the KO control samples from Tg and Dtt treated MEF experiments, and was reported again as the top cleavage target for an *Ire1α* target splice site. Although we have tested the RSR using human ENCODE datasets, our algorithm could also be easily extended for prediction of spliced regions for other species under any given parameter settings.

## Methods

### The RNA-Seq read sequence data

The mouse test data were downloaded from NCBI Gene Expression Omnibus (GEO) under the accession number GSE54631. Mouse embryonic fibroblast cells (MEF) that were heterozygous for *Ire1*α (*Ire1*α(+/−)) and cells which had *Ire1*α knocked out (*Ire1*α(−/−)) treated for 4 h with either 500nM Thapsigargin (Tg) or 1 mM Dithiothreitol (Dtt). Both RNA-Seq experiments are single end reads and had no experimental replicates performed.

The human test data are ENCSR000CUR which were downloaded from the ENCODE project (https://www.encodeproject.org/experiments/ENCSR000CUR/). The data were paired-end RNA-Seq experiments performed on human skin melanocytes primary whole cells (NHEM-M2) and sequenced using Illumina HiSeq 2000. There were two biological replicates (adult 52 years old and adult 55 years old) and no technical replicates used in this experiment.

### The reference genome for Read-Split-Run

We downloaded the mouse (mm9) and human (hg19) genome reference sequences from the University of California Santa Cruz (UCSC) genome browser (http://genome.ucsc.edu). We also downloaded respective UCSC gene files (knownGene.txt) from the UCSC genome browser. The splice junction file was created by setting the sequence entry on each side of the junction site to 4 bp shorter than the read length using a RNA-Seq software python script (getsplicefa.py) from ERANGE version 3.1 (http://woldlab.caltech.edu/~alim/RNAseq/). The original reference genome and splice junction site file were merged together to form an expanded genome.

### The algorithm of Read-Split-Run

**Fig. 5:**
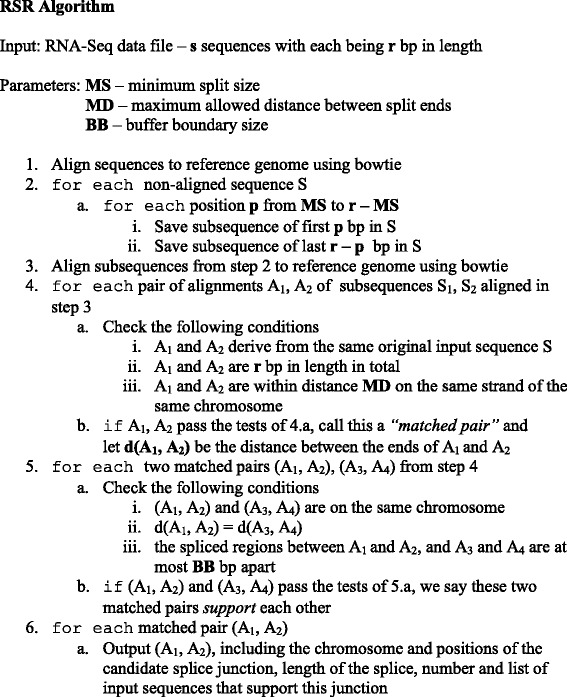
Pseudocode for Read-Split-Run algorithm. The junctions output by step 6 of the algorithm can optionally be restricted to those which are supported by some minimum number of sequences

We first recall the basic pipeline of the earlier work [10] before highlighting areas of improvement. Pseudocode is given for the pipeline in Fig. 5. The pipeline takes as input an RNA-seq file containing many short reads. The bowtie sequence aligner, version 1.0.1, [16] is first invoked, and unmapped reads are passed to the next stage of the pipeline as possible candidates resulting from the splicing. If a given non-aligned read sequence S did result from the splicing, the splice point could be at any position within S. The next stage of the algorithm splits each non-aligned sequence S into pairs (S_1_, S_2_) in all ways so that both parts are at least some minimum size (a parameter we denote MS, with a typical value between 8 and 1/3 of length of the original read sequences). Bowtie is invoked again, this time on each sub-sequence that resulted from splitting a non-aligned sequence from the first invocation of bowtie. Alignments of the sub-sequences are scanned for sub-sequences that were (i) derived as split pairs from the same original non-aligned read sequence, and (ii) aligned at positions on the same chromosome that are not too far apart (a parameter we denote MD, with a typical value of around 40,000). These conditions are consistent with a splicing event, and we save all pairs of alignments that satisfy the conditions, which we call “matched pairs”. The final stage of the pipeline scans all matched pairs to determine for each matched pair how many other matched pairs are likely a result of the same splice location; one matched pair “supports” another if the spliced region between the two ends is the same length and at a position on the same chromosome that is very close (a parameter we denote BB, with a typical value of between 2 and 5). The most interesting splice junctions are those with the highest number of matched pairs that support them.

The present work began by porting the previous pipeline from being written in Perl to C++ (compiled with g++ 4.8.3 using optimization parameter –O4 on Linux). Porting to C++ resulted in a speedup by a factor of roughly two to three. Other than the sequence alignment using bowtie, most of the running time in the pipeline is in comparing aligned sub-sequences to determine the set of matched pairs, and comparing matched pairs to determine which support each other. The previous pipeline compared all pairs in each step, resulting in a running time that is quadratic in the number of sub-sequences coming out of the second bowtie step. We improve this step drastically so that the running time is quadratic only in the number of sub-sequences that were derived from the same initial non-aligned read sequence (typically less than a few hundred, whereas there may be millions of sub-sequences coming out of the second bowtie step). We obtain a similar improvement in the step that scans matched pairs to determine which support each other.

### Methods of running Read-Split-Run on mouse MEF RNA-Seq data

The running parameter values employed for both Tg and Dtt samples are shown in Table 4. The steps of finding matched pairs and scanning matched pairs for supporting reads for Dtt samples were performed on a separate system running two Intel Xeon E5-2680 2.8Gz processors (a total of 20 cores and 40 hyperthreads). The highest running times are for tests with lower values of MS – this increases the number of sub-sequences that must be considered. A larger value for the length of initial read sequences also increases running time; the Dtt tests had higher running times because they consist of 77 bp (as opposed to 33 bp for Tg tests) and because the Dtt tests had roughly three times as many read sequences to begin with.

**Table 4:** The running parameter values employed for both Tg and Dtt samples

Sample	Minimum split size (MS)	Maximum candidate distance (MD)	Read mapping region boundary buffer size (BB)
Tg	8, 11, 12, 16	10000, 20000, 30000, 40000, 50000	1, 3, 5, 7, 9
DTT	11, 16, 20, 24	10000, 20000, 40000, 50000	3, 5, 7, 9

### Methods of running Read-Split-Run on human ENCODE RNA-Seq dataset

The phases: bowtie, splitting, and second step of bowtie were performed on same hardware mentioned above as the Dtt and Tg sets, whereas the RSR program was run on the “compute node,” described above. The parameters for this run were: MS - 33, MD - 50,000, and BB - 5. Before we could run the split-pairs portion of the pipeline, the output from bowtie (phase 2) had to be split into individual chromosomes so that they could fit into memory. Even in doing so, chromosome 14 had so much data (611Gb) that it could not be run. No junctions were identified on chromosome M.

### Comparison with other tools

We compared our RSR algorithm with a couple of other NGS based alternative splicing events detection tools (TopHat [5] and Alt Event Finder [6]). We applied these tools on RNA-Seq data from a mouse embryonic fibroblast (MEF) cell line to check which of these tools can identify and rank *Xbp1* as the top cleavage target and its presence and absence in *Ire1*^*+/−*^ and *Ire1*^*−/−*^ MEF samples and extended the analysis to the ENCODE RNA-Seq datasets. We ran TopHat v2.0.13 using options: −I 3000000, −g 10, --coverage-search, −microexon-search, to generate the “accepted_hits.bam” file for RNA-Seq data for each experiment condition from MEF cell line. Alt Event Finder v0.1 was ran by taking the “transcript.gtf” file generated from Cufflinks [17–20] and the “accepted_hits.bam” file generated by TopHat. Other metrics (i.e. running speed and usability) of these tools were also examined.

### A general linear model for RSR

**Fig. 6:**
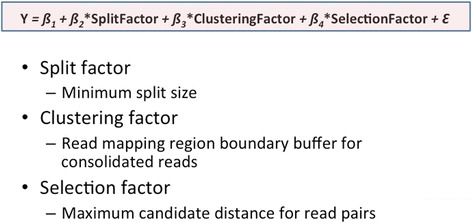
A modified General Liner Model for the Read-Split-Run algorithm

We proposed a modified General Linear Model (Fig. 6) for RSR. The variables (parameters) considered in the model are: minimum split size, maximum candidate distance, and read mapping region boundary buffer size. 80 test cases for Het and KO samples of both Tg and Dtt experiments were run on mouse MEF datasets to produce the General Linear Model equation.

## Abbreviations

BB, boundary buffer size; Dtt, Dithiothreitol; ENCODE, Encyclopedia of DNA Elements; ER, endoplasmic reticulum; GLM, General Linear Model; Het, heterozygote; KO, knock-out; MD, maximum candidate distance; MEF, mouse embryonic fibroblast; MS, minimum split size; RSR, Read-Split-Run; RSW**,** Read-Split-Walk; Tg, Thapsigargin; UPR, unfolded protein response

## Additional files

Additional file 1: Spliced regions identified by RSR for the Tg sample. This file contains all supplementary results for five test cases for the Tg sample. (XLSX 105 kb) Additional file 2: Spliced regions identified by RSR for the Dtt sample. This file contains all supplementary results for five test cases for the Dtt sample. (XLSX 2828 kb) Additional file 3: Statistics reported by RSR for 100 test cases for the Tg sample. This file contains all supplementary results for 100 test cases for the Tg sample. (XLSX 92 kb) Additional file 4: Statistics reported by RSR for 64 test cases for the Dtt sample. This file contains all supplementary results for 64 test cases for the Dtt sample. (XLSX 79 kb) Additional file 5: Total number of spliced regions identified by RSR in the human ENCODE RNA-Seq dataset. This file includes all supplementary results for number of supporting reads, splice length, range of supporting reads (spliced regions) identified by RSR in the human ENCODE RNA-Seq dataset. (XLSX 1411 kb)
